# Mental health, coping strategies, and help-seeking behaviour during the ongoing war in Ukraine: a cross-sectional study

**DOI:** 10.3389/ijph.2026.1610017

**Published:** 2026-08-25

**Authors:** Aneta Bednářová, Yana Kalymon, Pavol Mikula, Nataša Hlaváčová, Ingrid Obsuth, Dominika Jarčušková

**Affiliations:** 1 2nd Department of Psychiatry, Faculty of Medicine, University of Pavol Jozef Šafárik, Kosice, Slovakia; 2 Department of Anaesthesiology and Multidisciplinary Intensive Care, Penta Hospitals, Spišská Nová Ves, Slovakia; 3 Department of Social and Behavioural Medicine, Faculty of Medicine, Pavol Jozef Šafárik University, Košice, Slovakia; 4 Institute of Experimental Endocrinology, Biomedical Research Center, Slovak Academy of Sciences, Košice, Slovakia; 5 The University of Edinburgh School of Health in Social Science, Edinburgh, United Kingdom; 6 1st Department of Psychiatry, Faculty of Medicine, Pavol Jozef Šafárik University, Košice, Slovakia

**Keywords:** anxiety, coping strategies, depression, mental health, war

## Abstract

**Objectives:**

The war in Ukraine has had an impact on the population’s mental health. Displacement, economic instability, loss of loved ones, and shortages of basic resources have contributed to distress, including anxiety, depression, and post-traumatic stress disorder (PTSD).

**Methods:**

This study examined mental health symptoms and coping strategies in a sample of 100 Ukrainian adults (mean age = 35.5 ± 12.5; 73% female). Participants completed self-report questionnaires assessing symptoms of depression, anxiety, and PTSD, along with stressors, coping, and help-seeking behaviour. Logistic regression was used to identify predictors of mental health outcomes and service use.

**Results:**

Symptoms of depression were reported by 70% of participants, anxiety by 80%, and 31% met criteria for probable PTSD. Despite this, only 15% sought psychological help. Women were more likely than men to report anxiety and PTSD. Perceived stress significantly predicted mental health outcomes and was associated with a greater likelihood of help-seeking.

**Conclusion:**

These findings highlight the urgent need to improve access to psychological and psychiatric care in Ukraine.

## Introduction

On 24 February 2022, Russia’s invasion of Ukraine triggered one of the largest humanitarian crises and geopolitical disruptions in recent history. The war has led to widespread destruction, displacement, and loss of life, fundamentally disrupting everyday routines, social ties, and access to essential services. Ukrainians have faced involuntary migration, unemployment, financial instability, and the deaths of family members and loved ones. Constant threats from missile attacks, ongoing military activity, and the unpredictability of daily life have created an environment of sustained psychological insecurity.

Russia’s full-scale invasion of Ukraine in February 2022 triggered the largest displacement crisis in Europe since World War II. Internal displacement within Ukraine peaked at approximately 8.0 million people in May 2022 before declining to around 3.7 million by autumn 2025. At the same time, return migration has continued despite the ongoing war. By autumn 2025, an estimated 4.5 million people had returned to their places of origin, including more than 1.2 million Ukrainians who had returned from abroad, predominantly women and children. However, recent evidence indicates that return during an ongoing armed conflict is often associated with lower life satisfaction and poorer mental health, highlighting the psychological and structural challenges of reintegration into a country that remains at war [1]. Others remain in conflict zones, where exposure to violence, loss of housing, separation from family members, and economic hardship are common. In both cases, civilians are at increased risk of mental health problems. Importantly, the war is ongoing, and these stressors remain active, compounding over time and continuing to shape lived experience. Research in other conflict-affected populations has consistently documented elevated rates of depression, anxiety, and post-traumatic stress disorder (PTSD) [2, 3], yet there is comparatively little empirical data on how these problems are manifesting in the Ukrainian population during the current conflict.

Early assessments conducted shortly after the invasion suggested relatively low rates of mental health symptoms [4]. However, as the war continues, many individuals face chronic stressors and cumulative losses, suggesting the potential for worsening psychological outcomes over time [5]. Unlike post-conflict studies that evaluate trauma retrospectively, this study captures psychological distress during an ongoing crisis, providing insight into how mental health symptoms unfold under prolonged threat and instability.

Depression, anxiety, and PTSD are among the most prevalent mental health conditions in conflict-affected settings [2, 6]. Depression is typically characterised by persistent low mood, loss of interest or pleasure, fatigue, and concentration difficulties [7, 8], while anxiety disorders involve excessive worry and physiological hyperarousal that interfere with daily functioning [9–11]. PTSD is marked by intrusive memories, hypervigilance, emotional numbing, and avoidance behaviours following traumatic events [6, 12]. These disorders frequently co-occur and are often exacerbated in contexts where individuals experience repeated or multiple forms of adversity. Global epidemiological data suggest that women are at greater risk of developing such conditions, particularly PTSD and anxiety [9, 13].

While the psychological consequences of war are well recognised, the ways individuals respond to adversity vary widely. The transactional model of stress and coping was developed by Lazarus and Folkman [14] emphasises that emotional and behavioural responses are shaped not only by the nature of the stressor, but also by an individual’s appraisal of their capacity to cope. In addition, Hobfoll’s Conservation of Resources theory [15] suggests that psychological distress may intensify as individuals lose—or perceive a threat of losing—important personal, social, or material resources, a condition frequently observed in conflict settings. Coping strategies, including emotion-focused responses, avoidance, or active problem-solving, may mitigate or amplify the effects of stress, yet in low-resource environments, access to professional support may be limited, and stigma or lack of awareness may deter help-seeking [16, 17].

This study aims to examine the mental health impact of the ongoing Russo-Ukrainian war on a sample of Ukrainian adults. Specifically, it investigates the prevalence of depression, anxiety, and PTSD; identifies commonly reported stressors and coping strategies; explores sex differences in symptom presentation; and examines associations between perceived stress and psychological outcomes.

## Methods

### Participants and procedure

A total of 100 participants were recruited from Ukraine using online convenience sampling through social media platforms and community networks. Eligibility criteria included being 18 years or older, residing in Ukraine or having been displaced due to the war, and fluency in Ukrainian. All participants completed an online questionnaire voluntarily and anonymously via Google Forms. Data were collected from January 2025 to September 2025. Before beginning the survey, participants provided electronic informed consent, acknowledging their understanding of the study’s purpose and their right to withdraw at any time. The study was approved by the Ethics Committee of the Faculty of Medicine at Pavol Jozef Šafárik University in Košice (Protocol No. 6N/2025) and conducted in accordance with the Declaration of Helsinki and its later amendments. In addition to mental health variables, participants provided basic sociodemographic information, including age and sex.

### Measures

Depressive symptoms were assessed using the Beck Depression Inventory-II (BDI-II) [18], a 21-item self-report scale measuring depressive symptoms over the past 2 weeks. Items are rated from 0 to 3, with total scores ranging from 0 to 63. Severity was classified as minimal (0–13), mild (14–19), moderate (20–28), or severe (29+). The BDI-II shows high internal consistency (α = 0.86–0.92). Cronbach’s alpha in this sample was 0.91.

Anxiety symptoms were measured using the Beck Anxiety Inventory (BAI) [19], which includes 21 items rated on a scale of 0–3. Total scores range from 0 to 63. While the original manual does not define cut-offs, we applied widely used thresholds: minimal (0–7), mild (8–15), moderate (16–25), and severe (26+), following Julian [20]. Internal consistency is typically high (α = 0.85–0.94). Cronbach’s alpha in this sample was 0.92.

PTSD symptoms were assessed using the PTSD Checklist for DSM-5 (PCL-5) [21], a 20-item measure rated on a 5-point scale (0–4), yielding scores from 0 to 80. A cut-off of 33 was used to indicate probable PTSD. The PCL-5 demonstrates excellent internal consistency (α > 0.90). Cronbach’s alpha in this sample was 0.96.

Participants also answered multiple-choice questions developed by the researchers to assess war-related and general stressors (e.g., separation from family, financial insecurity), coping strategies, and use of psychological or psychiatric support. Coping strategies included both adaptive responses (e.g., talking with loved ones, engaging in hobbies) and maladaptive behaviours (e.g., overeating, medication use).

The questionnaire items were developed by a multidisciplinary panel of four clinicians and researchers in psychiatry and psychology based on clinical experience and relevant literature. The preliminary version of the questionnaire was reviewed by the panel to assess face and content validity and was pilot-tested in a subsample of 60 participants before the main analyses. Based on the pilot testing, the wording of several items and response options was refined to improve clarity and comprehensibility. As the questionnaire was developed specifically for the purposes of the present study, no formal psychometric validation was performed.

A cumulative war-exposure index was created *a priori* by summing 11 dichotomous (yes/no) items assessing war-related exposures and experiences. These included displacement from one’s place of permanent residence, loss of housing or property, loss of a loved one, presence in or near an active combat zone, current or past military service, participation in combat, war-related physical or mental health problems, having been under occupation, and having a loved one affected by the war (e.g., serving in the armed forces, living in a war zone, being under occupation, or in captivity). Higher scores indicated greater cumulative war exposure, with possible scores ranging from 0 to 11.

### Statistical analyses

Data were analysed using Stata Special Edition, Version 13.1 (StataCorp LP, College Station, TX). The Shapiro–Wilk test was used to assess the normality of continuous variables. Categorical variables were reported as frequencies and percentages.

To examine the associations between mental health symptoms and predictors (e.g., stressors, sex, coping strategies), ordinal logistic and binary logistic regression were employed. Multivariable logistic regression models for the three primary outcomes were performed. We deliberately specified a parsimonious adjustment set — age, sex, and a cumulative war-exposure index — because the number of cases for the least prevalent outcome (probable PTSD, n = 31) constrains the number of predictors the data can support: at an events-per-variable ratio of approximately 10, three covariates were the maximum that yielded stable estimates.

Results were reported using odds ratios (OR), standard errors (SE), 95% confidence intervals (CI), and associated p-values. The chi-squared test was used to evaluate relationships between categorical variables. Statistical significance was set at p < 0.05. The dataset was complete: all participants had complete data on all variables used in the reported analyses, so no cases were excluded and no imputation was required.

## Results

A total of 100 participants completed the survey. Of these, 27% were male (n = 27) and 73% were female (n = 73), with a mean age of 35.5 years (SD = 12.6; range = 18–69). The majority (89%) reported experiencing significant stress during the war.

**FIGURE 1:**
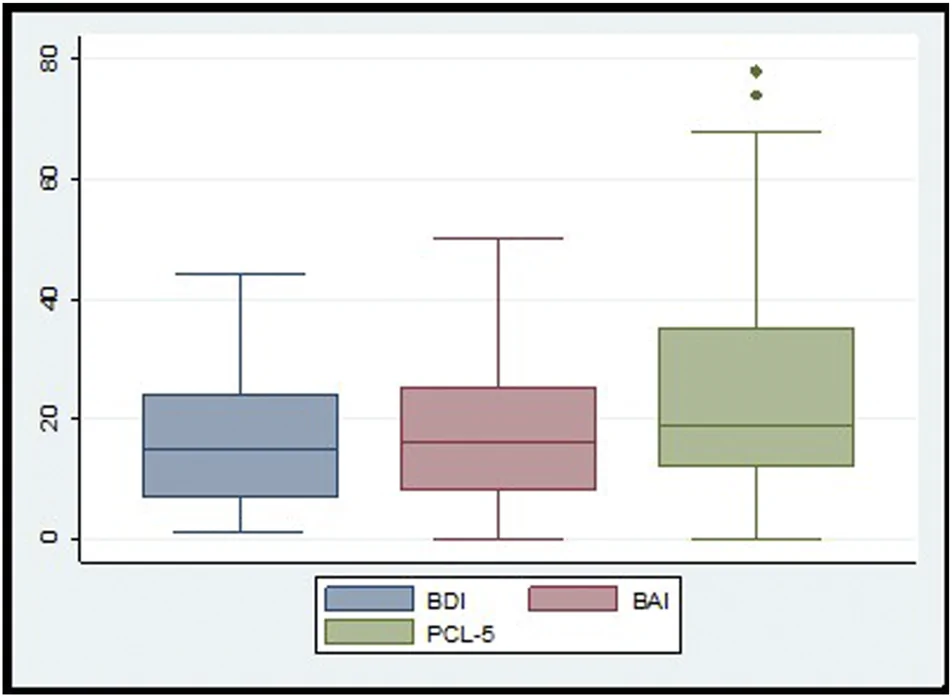
Scores on the BDI, BAI and PTSD among participants. Note: BDI- Beck depression index, BAI- Beck anxiety index, PCL-5- PTSD Checklist for DSM-5 (Košice, Slovakia. 2025).

According to the Beck Depression Inventory-II, 30% of participants had minimal depressive symptoms, 30% had mild symptoms, 27% had moderate symptoms, and 13% met the threshold for severe depression. The mean BDI-II score was 16.83 (SD = 10.48). Anxiety symptoms, as measured by the Beck Anxiety Inventory, were also widespread: 20% of participants reported minimal symptoms, 27% mild, 29% moderate, and 24% severe anxiety. The mean BAI score was 18.49 (SD = 11.96). Based on the PCL-5, 31% of participants met criteria for probable PTSD, while 69% did not. The mean PTSD Checklist score was 24.61 (SD = 18.15) (see Figure 1).

Despite the high prevalence of symptoms, only 15% of participants reported seeking psychological help. Sex differences emerged in the prevalence of anxiety and PTSD, with females more likely to report higher symptom levels. In univariable regression analyses, female sex was significantly associated with higher odds of anxiety (OR = 2.84, 95% CI: 1.26–6.54, p = 0.01) and probable PTSD (OR = 3.30, 95% CI: 1.13–12.16, p = 0.04), but not depressive symptoms (OR = 2.16, 95% CI: 0.95–5.01, p = 0.06).

The proportional-odds assumption was tested using the Brant test for the univariable models (BDI, BAI); the assumption held for both outcomes (BDI: χ^2^ = 0.39, df = 2, p = 0.82; BAI: χ^2^ = 0.76, df = 2, p = 0.68).

Further analyses examined associations between depressive symptoms and stressors. Participants with depressive symptoms were significantly more likely to report feeling stressed (OR = 16.19, 95% CI: 3.28–79.8, p = 0.001), attributing their stress to the war (OR = 4.27, 95% CI: 1.71–10.66, p = 0.002), and experiencing mental health problems due to the war (OR = 5.06, 95% CI: 1.60–16.02, p = 0.006). They were also more likely to report separation from family (OR = 2.42, 95% CI: 1.01–5.81, p = 0.047) and fear of the future abroad (OR = 8.18, 95% CI: 1.02–65.00, p = 0.047) as major stressors. Regarding coping, those with depressive symptoms were more likely to use maladaptive strategies such as eating more sweets (OR = 3.47, 95% CI: 1.19–10.11, p = 0.023) and less likely to spend time with loved ones (OR = 0.37, 95% CI: 0.16–0.89, p = 0.026) (see Table 1).

**TABLE 1:** Factors associated with depressive and anxiety symptoms and probable PTSD (Košice, Slovakia. 2025).

​	OR	SE	95% CI	p
Depressive symptoms (BDI)				
Felt stressed	16.19	13.18	3.28–79.8	0.001
The main reason for your stress and nervousness- war with Russia	4.27	1.99	1.71–10.66	0.002
Mental health problems due to the war	5.06	2.98	1.6–16.02	0.006
Aspects of war that are causing (have caused) stress or nervousness - separation from relatives and family	2.42	1.08	1.01–5.81	0.047
Aspects of war that are causing (have caused) stress or nervousness- fear of what awaits abroad	8.18	8.65	1.02–65.00	0.047
Coping strategies- eating more sweet/tasty food	3.47	1.89	1.19–10.11	0.023
Coping strategies- spending time with loved ones	0.37	0.17	0.16–0.89	0.026
Coping strategies- taking medication to calm down	9.62	10.14	1.22–75.95	0.032
Anxiety symptoms (BAI)				
Felt stressed	19.25	14.16	4.55–81.35	<0.001
The main reason for your stress and nervousness- war with Russia	6.02	3.17	2.15–16.88	0.001
The main reason for your stress and nervousness- work/study	6.33	4.93	1.38–29.08	0.018
Mental health problems due to the war	6.33	4.93	1.38–29.08	0.018
Aspects of war that are causing (have caused) stress or nervousness - separation from relatives and family	3.21	1.71	1.13–9.14	0.028
Coping strategies- hobbies	0.34	0.17	0.13–0.93	0.034
Probable PTSD				
The main reason for your stress and nervousness- financial problems	2.65	1.23	1.06–6.60	0.037
The main reason for your stress and nervousness- unemployment	10.22	11.66	1.09–95.64	0.042
Mental health problems due to the war	4.67	2.16	1.89–11.56	<0.001
Physical health problems due to war	3.17	1.73	1.09–9.24	0.034
Coping strategies- taking medication to calm down	3.69	1.98	1.29–10.58	0.015
Coping strategies- psychological counselling/psychotherapy	4.36	2.54	1.39–13.66	0.011

A similar pattern emerged for anxiety. Participants with anxiety symptoms (n = 80) reported higher levels of perceived stress (OR = 19.25, 95% CI: 4.55–81.35, p < 0.001), stress due to war (OR = 6.02, 95% CI: 2.15–16.88, p = 0.001), and work or study-related stress (OR = 6.33, 95% CI: 1.38–29.08, p = 0.018). They were also more likely to report mental health impacts due to the war (OR = 6.33, 95% CI: 1.38–29.08, p = 0.018) and separation from relatives (OR = 3.21, 95% CI: 1.13–9.14, p = 0.028). Anxiety was also associated with a lower likelihood of engaging in hobbies (OR = 0.34, 95% CI: 0.13–0.93, p = 0.034) (see Table 1).

For PTSD, participants with symptoms of probable PTSD (n = 31) were more likely to report financial problems (OR = 2.65, 95% CI: 1.06–6.60, p = 0.037), unemployment (OR = 10.22, 95% CI: 1.09–95.64, p = 0.042), physical health problems related to war (OR = 3.17, 95% CI: 1.09–9.24, p = 0.034), and use of medication to calm down (OR = 3.69, 95% CI: 1.29–10.58, p = 0.015). They were also more likely to report receiving psychological counselling or psychotherapy (OR = 4.36, 95% CI: 1.39–13.66, p = 0.011) (see Table 1).

**FIGURE 2:**
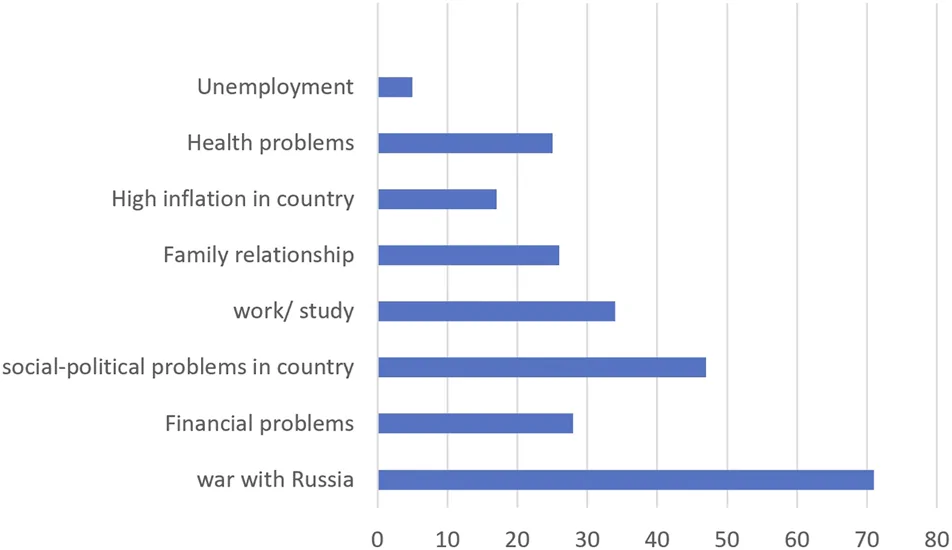
General sources of stress and nervousness reported by participants (Košice, Slovakia. 2025).

**FIGURE 3:**
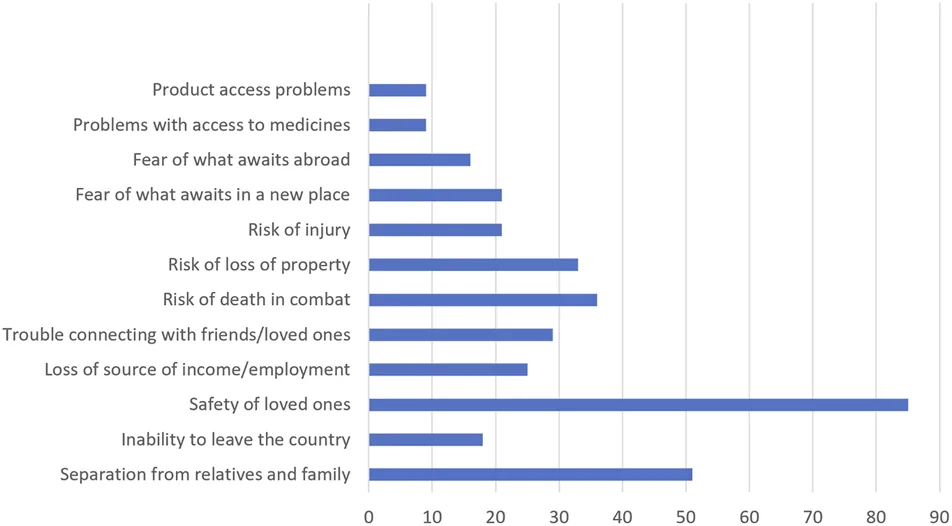
Specific war-related stressors reported by participants (Košice, Slovakia. 2025).

Across the full sample, the most frequently reported sources of stress were war-related: concerns about the safety of loved ones, separation from family, and uncertainty about the future (Figure 2). In terms of coping strategies, participants most reported communicating with loved ones, engaging in hobbies or work, watching television, listening to music, or browsing the internet (Figure 3). A minority reported using psychological services or medication.

The war with Russia was the most frequently reported source of stress (Figure 2). All participants were additionally asked about specific war-related stressors, regardless of whether they identified the war as their main source of stress; these results are presented in Figure 3.

**FIGURE 4:**
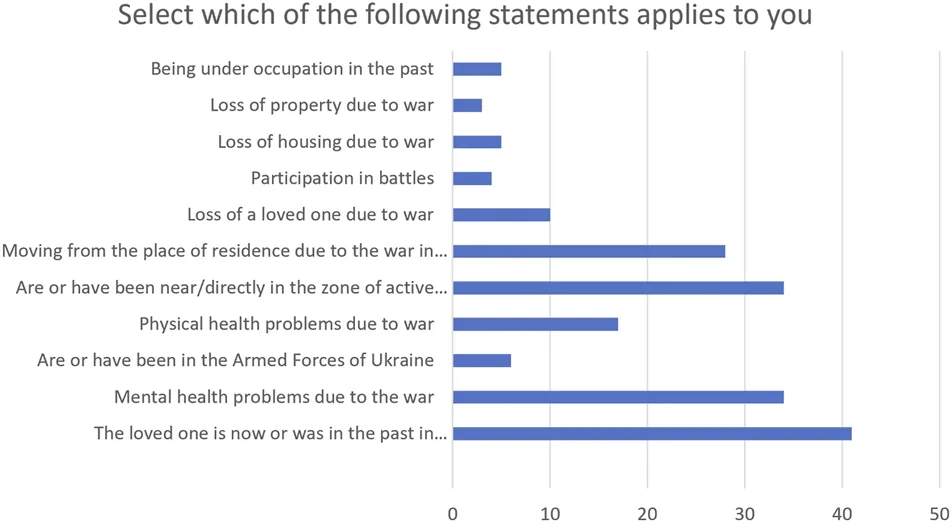
War-related experiences reported by participants (Košice, Slovakia. 2025).

Participants also indicated specific life disruptions experienced as a result of the war. The most commonly reported experiences included having a loved one currently or previously in a war zone (43%), experiencing mental health problems due to the war (38%), being near or directly in active conflict zones (36%), and being displaced from their place of residence (34%). Other frequently reported experiences included physical health problems, loss of a loved one, or having served in the armed forces. These findings illustrate the breadth and depth of war-related exposure among the sample (see Figure 4).

**FIGURE 5:**
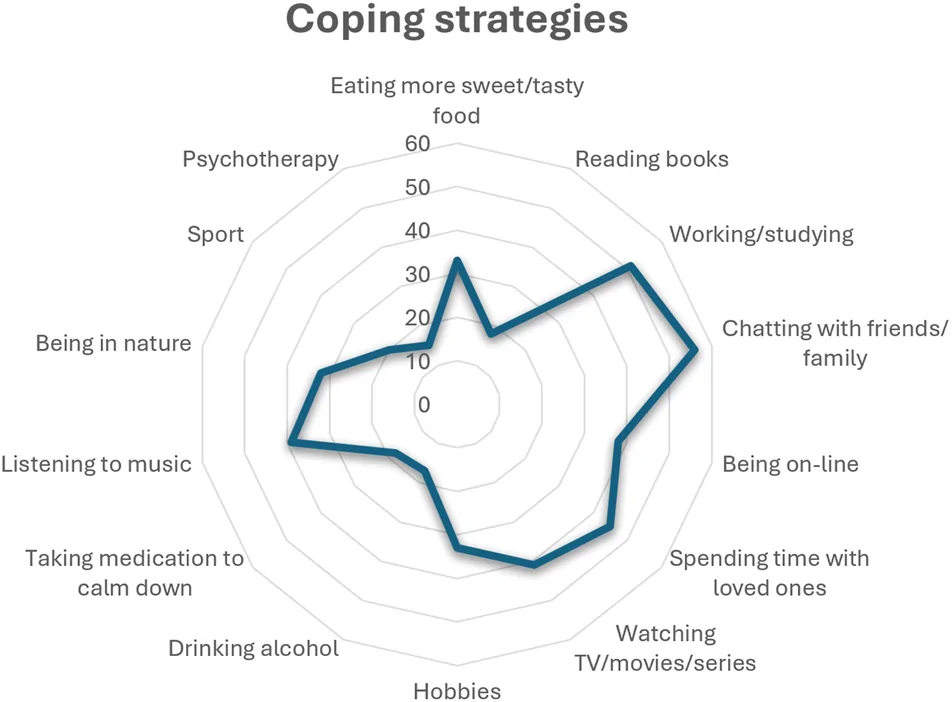
Coping strategies reported participants by participants (Košice, Slovakia. 2025).

Participants also reported a wide range of coping strategies used to manage their current situation, encompassing both adaptive and maladaptive behaviours. The most commonly reported strategies included maintaining communication with friends and family, engaging in work or study, spending time online, and consuming comforting or sweet foods. Distraction techniques such as watching television, listening to music, and browsing the internet were also frequently endorsed. While fewer participants engaged in more formal mental health support, participants with probable PTSD were significantly more likely to report the use of psychotherapy (OR = 4.36, 95% CI: 1.39–13.66, p = 0.011) or medication to calm down (OR = 3.69, 95% CI: 1.29–10.58, p = 0.015). A visual summary of coping strategies is presented in Figure 5.

### Multivariable regression analyses

Multivariable regression analyses were performed to examine whether the observed associations persisted after adjustment for potential confounders. Three separate models were fitted, one for each primary outcome, adjusting for age, sex, and the cumulative war-exposure index. After adjustment, female sex remained independently associated with higher depression severity (aOR = 2.62, 95% CI: 1.08–6.34, p = 0.032). Neither the cumulative war-exposure index (aOR = 1.26, 95% CI: 0.92–1.72, p = 0.147) nor age (aOR = 0.97, 95% CI: 0.94–1.01, p = 0.101) was independently associated with depressive symptoms.

Similarly, female sex was independently associated with higher anxiety severity (aOR = 2.82, 95% CI: 1.19–6.67, p = 0.018). In addition, the cumulative war-exposure index was independently associated with greater anxiety severity (aOR = 1.37, 95% CI: 1.03–1.83, p = 0.032), whereas age was not significantly associated with anxiety (aOR = 1.01, 95% CI: 0.98–1.04, p = 0.699).

For probable PTSD, female sex also remained an independent predictor after adjustment (aOR = 3.68, 95% CI: 1.09–12.42, p = 0.036). Neither the cumulative war-exposure index (aOR = 1.26, 95% CI: 0.90–1.76, p = 0.176) nor age (aOR = 0.99, 95% CI: 0.95–1.03, p = 0.587) was significantly associated with probable PTSD.

Overall, female sex remained independently associated with all three mental health outcomes after adjustment. The cumulative war-exposure index was independently associated only with anxiety severity, whereas age was not significantly associated with any of the outcomes. The proportional-odds assumption was evaluated using the Brant test for the adjusted ordinal logistic regression models. The assumption was met for both depression (χ^2^ = 3.11, df = 6, p = 0.80) and anxiety (χ^2^ = 2.96, df = 6, p = 0.81).

## Discussion

This study highlights the substantial psychological toll of the Russo-Ukrainian war, with participants reporting high levels of depressive symptoms (70%), anxiety symptoms (80%), and probable PTSD (31%) amid an ongoing and unresolved armed conflict. These rates are substantially higher than global prevalence estimates for these disorders in the general population [6, 11, 22]. Other studies of war-affected Ukrainians report a similar psychological burden. Although complex post-traumatic stress disorder (CPTSD) and PTSD represent distinct constructs, Hutul et al. (2025) found that 33.4% of respondents likely exhibited diagnostic symptoms of CPTSD [23]. Similarly, research on displaced Ukrainians in host countries has documented elevated psychological distress, with modifiable post-migration factors—such as access to information, psychological support, and skills-matched employment—emerging as key correlates of mental health and adaptive coping [24]. Unlike many trauma studies conducted post-event, these data reflect psychological distress occurring during a period of continuing instability, threat, and loss, where safety, livelihood, and daily routines remain disrupted.

Sex differences were observed in the prevalence of anxiety and PTSD, with female participants more likely to report elevated symptoms. This finding aligns with prior epidemiological research [9, 11] and may partly reflect differences in caregiving responsibilities, although these mechanisms were not assessed in the present study. No statistically significant difference in depressive symptoms was observed between men and women, which is not fully consistent with patterns reported in global epidemiological studies [13]. This finding should be interpreted cautiously given the characteristics of the study sample.

Participants reported a range of war-related stressors, including displacement, financial hardship, health problems, separation from family, and uncertainty about the future. These experiences, and their close association with mental health symptoms, can be understood through the lens of Lazarus and Folkman’s transactional model of stress and coping [14], which proposes that individuals’ appraisals of their stress and perceived coping resources strongly influence psychological outcomes. In addition, the Conservation of Resources theory [15] highlights the role of actual or threatened loss of personal, social, and material resources in generating stress. In the context of war, where access to safety, relationships, and stability is routinely disrupted, distress may emerge not only from trauma exposure but also from the prolonged erosion of protective resources.

The data also support cumulative trauma and polyvictimisation frameworks [25], as participants with symptoms of depression and anxiety were more likely to report multiple intersecting stressors, including displacement, financial strain, and concern for family members in war zones. PTSD symptoms, in particular, were associated with unemployment, physical health issues, and the experience of resource deprivation, reinforcing the idea that psychological burden may be linked to a combination of trauma exposure and broader structural vulnerabilities. Crucially, these stressors are ongoing, not past; the conflict continues to evolve, and the long-term psychological consequences of continued exposure remain uncertain. This further underscores the need for longitudinal monitoring of mental health outcomes in conflict-affected populations. Importantly, the capacity of mental health services in Ukraine may be further constrained by the fact that providers themselves operate within the same traumatic reality as their clients. A recent qualitative study of Ukrainian psychotherapists working in war zones identified compassion fatigue and secondary traumatization as the predominant negative occupational consequences of this prolonged shared traumatic reality. At the same time, active coping, social support, and the transgenerational transmission of empowering messages emerged as key protective factors [26].

Coping strategies varied across the sample. Adaptive responses such as connecting with friends and family, engaging in work or hobbies, and using distraction techniques were commonly reported. However, participants experiencing more severe symptoms were also more likely to engage in potentially maladaptive coping behaviours, including increased food consumption or use of medication to calm down. Only 15% of participants reported seeking psychological support, an especially low rate given the high burden of symptoms observed. These findings can be better understood by distinguishing between adaptive and avoidance-based coping strategies. Adaptive strategies observed in our sample, such as maintaining contact with loved ones and engaging in work or hobbies, reflect problem- and emotion-focused coping, which has consistently been associated with better psychological outcomes among war-affected Ukrainians. Among Ukrainian mental health professionals, active coping and social support emerged as key protective factors [26]. Similarly, among Ukrainian refugees in Poland, problem- and emotion-focused coping strategies predominated over avoidance and were facilitated by access to psychological assistance [24]. In contrast, the behaviours associated with greater symptom severity in our sample—namely increased consumption of sweet foods and using medication to calm down—reflect avoidance-based coping. Although such responses may provide temporary emotional relief, they do not address the underlying stressor and may contribute to the persistence of psychological distress. This interpretation is supported by comparative evidence showing that avoidant coping was positively associated with psychological distress among both Ukrainian and Polish college students [27]. Likewise, substance use as a coping strategy has been linked to higher levels of depressive symptoms among Ukrainian students, whereas health-promoting behaviours were associated with better mental health outcomes [28]. Notably, some frequently reported coping strategies in our sample, such as watching television or browsing the internet, appear to occupy an intermediate position. Distraction may be adaptive in the short term when stressors are largely uncontrollable, as is often the case during armed conflict. However, prolonged reliance on distraction at the expense of more approach-oriented coping strategies may become maladaptive as exposure to war-related stress continues. Therefore, interventions aimed at promoting approach-oriented and acceptance-based coping, including mindfulness-based interventions adapted for war-affected populations [29], may be particularly valuable in supporting long-term psychological adjustment.

This pattern aligns with findings from other studies of Ukrainians during the current conflict. For example, Chudzicka Czupała et al. found that 52.5% of Ukrainians stated they would not seek psychological help [17]. Similarly, Schäfer et al. reported that many Ukrainians may not recognise war-related psychological distress as requiring professional intervention, often believing that the resolution of war itself would resolve psychological suffering [16]. These findings suggest that psychological issues may be deprioritised in favour of immediate physical survival needs, or that stigma and limited service availability restrict access. In an ongoing war context, individuals may also perceive mental health support as premature or inaccessible while threats persist. Conventional mental health services, such as screening by primary care providers, psychoeducation, or community-based counselling, are difficult to implement in areas affected by displacement and insecurity. Therefore, such interventions could be supplemented with low-threshold, mobile, or online psychological support to meet the urgent needs of people affected by armed conflict [16].

Taken together, these findings underscore the urgent need to strengthen and adapt mental health services in Ukraine. The use of validated instruments across multiple domains, and the inclusion of coping and help-seeking data, are strengths of this study, providing a multifaceted view of psychological functioning under wartime conditions. However, limitations should be acknowledged. The cross-sectional design limits causal inference, and the use of convenience sampling and online recruitment may restrict the generalisability of findings. The sample was also predominantly female, which may have influenced the observed prevalence patterns and reduced the precision of sex-specific comparisons. Therefore, findings related to sex differences should be interpreted with caution. Furthermore, the relatively small sample size and convenience sampling approach limit the representativeness of the study population and reduce the generalizability of the findings to the broader Ukrainian population. Self-report instruments may be affected by response bias and social desirability bias, and were not revalidated for use in the specific cultural context of wartime Ukraine. Additionally, the lack of a pre-war baseline prevents conclusions about changes over time.

The study was exploratory in nature, and no *a priori* sample size or power calculation was performed. The sample of 100 participants — and, in particular, the small number of cases for lower-prevalence outcomes such as probable PTSD (n = 31) — limits statistical power and the stability of the logistic regression estimates. This is reflected in the wide confidence intervals for several associations, whose lower bounds lie only marginally above the null. Such estimates are imprecise and should be regarded as hypothesis-generating rather than confirmatory. Given the number of univariable comparisons performed, the possibility of Type I error (spurious associations arising by chance) cannot be excluded, and the limited power also raises the risk of Type II error, whereby genuine associations may have gone undetected. These findings should therefore be interpreted with caution and considered exploratory. Confirmation in larger, adequately powered, and more representative studies with pre-specified hypotheses is needed before broader conclusions can be drawn.

Future research should prioritise longitudinal designs that track psychological trajectories and adaptation during conflict and beyond. As the war continues, psychological needs may shift or intensify, requiring flexible and sustained mental health responses. Larger, adequately powered studies using more representative sampling strategies, including harder-to-reach or displaced populations, are needed to improve the external validity and generalizability of future findings.

### Conclusion

This study provides evidence of the substantial psychological burden associated with the ongoing Russo-Ukrainian war among conflict-affected civilians. High levels of depressive symptoms, anxiety symptoms, and probable PTSD were observed, with women and individuals exposed to multiple overlapping stressors appearing particularly vulnerable. Although adaptive coping strategies were commonly reported, the low use of professional psychological services suggests persistent structural and attitudinal barriers to mental healthcare. These findings highlight the need for accessible, scalable, and trauma-informed mental health services that can be delivered under conditions of prolonged conflict and displacement. The findings should be interpreted with caution because of the relatively small sample size and the predominance of female participants. Further large-scale, representative longitudinal studies are needed to confirm these findings and better understand the long-term mental health consequences of the war.

## Data Availability

The datasets generated and analysed during the current study are not publicly available due to confidentiality concerns (in the informed consent, we have made a commitment to the participants to publish only the general and group results of the study).
